# Comparison of injective related reactions following ofatumumab and ocrelizumab in patients with multiple sclerosis: data from the European spontaneous reporting system

**DOI:** 10.3389/fneur.2024.1383910

**Published:** 2024-06-27

**Authors:** Cristina Scavone, Antonietta Anatriello, Isabella Baccari, Andrea Cantone, Daniele Di Giulio Cesare, Francesca Futura Bernardi, Ornella Moreggia, Valerio Liguori, Vincenzo Andreone, Giorgia Teresa Maniscalco, Annalisa Capuano

**Affiliations:** ^1^Department of Experimental Medicine, University of Campania “Luigi Vanvitelli”, Naples, Italy; ^2^Regional Center of Pharmacovigilance and Pharmacoepidemiology of Campania Region, Naples, Italy; ^3^Multiple Sclerosis Regional Center, "A. Cardarelli" Hospital, Naples, Italy; ^4^Directorate-General for Health Protection, Campania Region, Naples, Italy; ^5^Neurological Clinic and Stroke Unit, "A. Cardarelli" Hospital, Naples, Italy

**Keywords:** Eudravigilance, infusion reactions, injective reactions, multiple sclerosis, ocrelizumab, ofatumumab

## Abstract

**Introduction:**

In 2021 ofatumumab, a recombinant human anti-CD20 monoclonal antibody (mAb) already authorized for the treatment of chronic lymphocytic leukemia, received the marketing approval for the treatment of relapsing forms of multiple sclerosis (MS). Differently from ocrelizumab, that is administered intravenously, ofatumumab if the first anti-CD20 mAb to be administered subcutaneously without a premedication.

**Methods and objectives:**

In this study we aimed to describe and compare the main characteristics of Individual Case Safety Reports (ICSRs) describing the occurrence of Injective Related Reactions (IRRs) following the treatment with ocrelizumab and ofatumumab reported in the Eudravigilance (EV) database during years 2021–2023.

**Results:**

A total of 860 ICSRs with either ofatumumab and ocrelizumab as suspected drug were retrieved from Eudravigilance, of which 51% associated with ofatumumab and 49% with ocrelizumab. The majority of patients who experienced IRRs following ocrelizumab belonged to the age group of 18–64 years (73%), while the age-group was mostly not specified (55%) in ICSRs reporting ofatumumab as suspected. The distribution of gender was almost similar in the two groups, with the majority of ICSRs related to female patients. “Pyrexia” was the Preferred Term (PT) most reported for ofatumumab, while “Infusion related reaction” were more frequently reported with ocrelizumab. Premedication drugs were reported in 148 ICSRs. Out of 89 ICSRs for which the Time to Event (TTE) was calculated, 74 reported IRRs that occurred the same day of the drug administration.

**Discussion:**

Based on the results of this study, although a risk of ofatumumab-induced IRRs cannot be excluded, it should be considered as manageable considering that the drug seems to be mostly associated with the occurrence of fever. Thus, it is important to continue to closely monitor the use of these in clinical practice to improve the knowledge on their long-term safety.

## 1 Introduction

The pharmacological armamentarium of multiple sclerosis (MS) has improved thanks to the approval of new disease modifying therapies (DMTs) acting through different biological mechanisms (1–3). Considering the key role of the immune system in MS, high-efficacy DMTs that target the immune system have emerged over the past decades, such as anti-CD20 monoclonal antibodies (mAbs) acting through the depletion of CD20+ B and CD20+ T cells (4). Anti-CD20 mAbs class currently includes ocrelizumab (approved by the EMA in 2018), rituximab (not officially approved for the treatment of MS), ublituximab (approved by the EMA in 2023) and ofatumumab (approved in 2021) (5, 6). These drugs mainly differ for the route of administration. Indeed, while ocrelizumab, rituximab, ublituximab are injected intravenously and require patients to receive a premedication in order to avoid injection-related reactions (IRRs) (7, 8), ofatumumab is meant to be administered by subcutaneous injection by the patients themselves, without any premedication since ofatumumab-induced IRRs are manageable (9). In addition, as reported in the product’s European Public Assessment Report (EPAR), the pre-treatment is not recommended also because steroids may reduce the frequency of fever, myalgia, chills, and nausea but conversely increase the occurrence of flushing, chest discomfort, hypertension, tachycardia and abdominal pain (9). The subcutaneous administration is made with an auto-injector pen that is administered at four-week intervals with the first three doses delivered on days 1, 8, and 15. Subcutaneous ofatumumab has been developed to reduce the occurrence of specific adverse events (AEs) like IRRs, considering that the subcutaneous route allows for a slower and more controlled absorption of the medication into the body, minimizing the risk of systemic reactions (4). In this regard, the indirect comparison between subcutaneous ofatumumab and ocrelizumab, showed that ofatumumab was less likely associated with IRRs (10, 11). Data from clinical studies showed that these events were generally mild-to-moderate in severity and no life-threatening IRRs were observed. The most common IRRs’ symptoms were fever, headache, chills, fatigue, erythema/redness and pain. Data from the post-marketing experience highlighted 6 serious IRRs, including a case of anaphylaxis (12, 13). Lastly, the results of the phase 2 MIRROR study reported that the most common AEs associated with ofatumumab were IRRs, mainly classified as not serious (serious IRRs occurred in 3 patients out of 121 patients treated with the drug, including one patient who experienced a cytokine-release syndrome within hours of the first ofatumumab injection) (14). As recently reported by Ovchinnikov and Findling, the availability of subcutaneous anti-CD20 mAb represents a huge advantage and a simplification of therapy for many patients (15). Notwithstanding few disadvantages need to be considered, such as for instance the possibility of a reduced patient compliance and the occurrence of ADRs at the injection site.

Considering the recent marketing approval of ofatumumab, the main purposes for which a subcutaneous formulation was developed and that recommendations on premedication to prevent IRRs are different between subcutaneous and intravenous anti-CD20 mAbs’ formulations, we aim to describe and compare the main characteristics of IRRs associated with ocrelizumab and ofatumumab using data from the European spontaneous reporting system database, EudraVigilance (EV).

## 2 Methods

### 2.1 Data source

ICSRs reporting ofatumumab and ocrelizumab as suspected drugs were retrieved from the EV website. This is the European spontaneous reporting system, publicly accessible at www.adrreports.eu, which allows the collection, management and analyses of ICSRs related to medicines or vaccines authorized or being studied in clinical trials in the European Economic Area (EEA). The EV is managed by the European Medicines Agency (EMA).

In EV, AEs are coded using event-related information according to the Medical Dictionary for Regulatory Activities (MedDRA). MedDRA was developed by the International Council for Harmonization of Technical Requirements for Pharmaceuticals for Human Use (ICH) and it is a hierarchical dictionary that is utilized to code signs, symptoms and diagnoses, surgical and medical procedures, investigations, medical/social history and therapeutic indications and for the registration, documentation and safety monitoring of products during the marketing authorization process (16–18).

### 2.2 Selection of ICSRs

**Figure 1:**
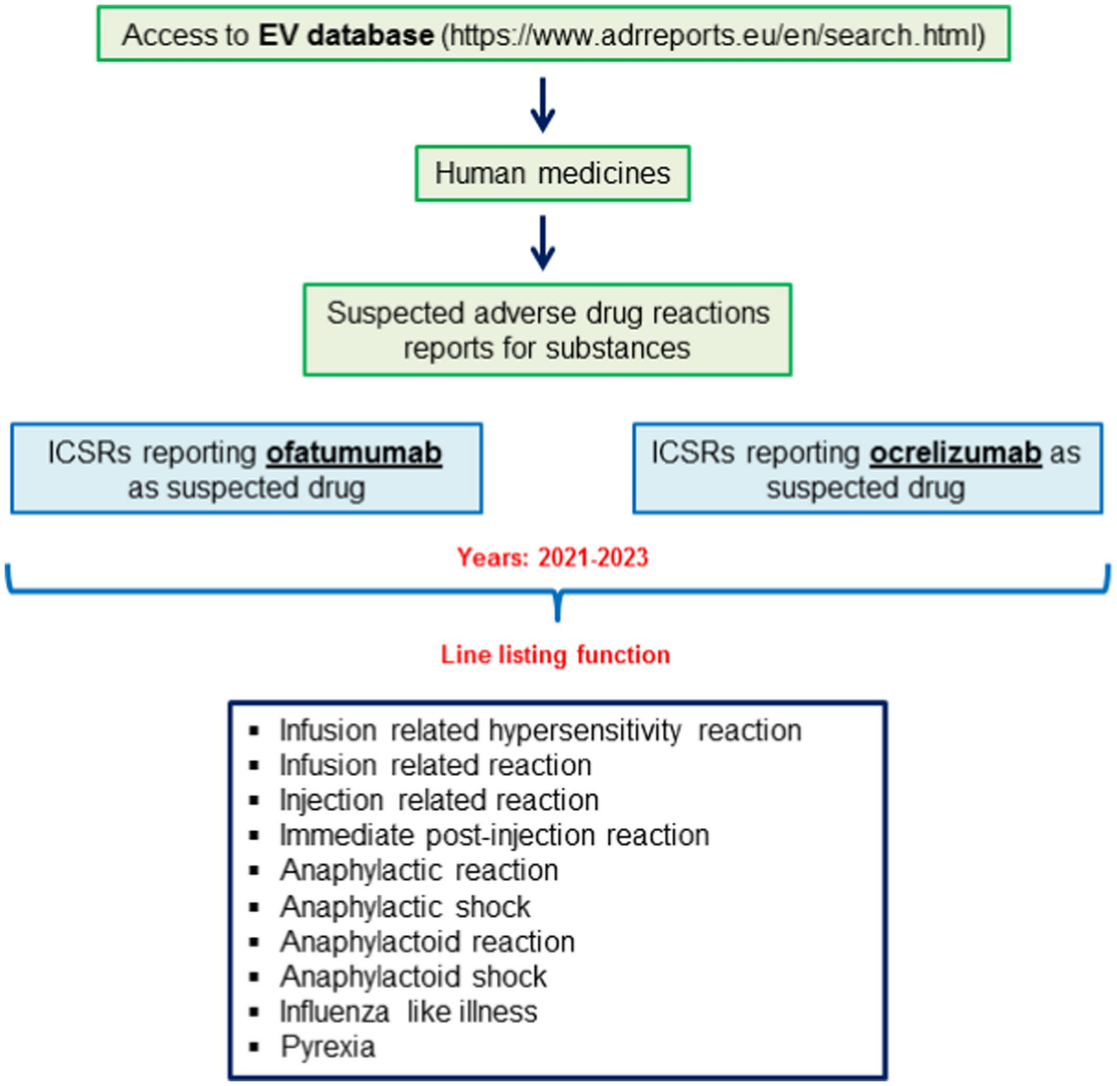
Selection of individual case safety reports (ICSRs) reporting cases of ofatumumab-and ocrelizumab-induced infusion related reactions (IRRs) from the EudraVigilance database.

Steps that were taken to access to ICSRs reporting ofatumumab and ocrelizumab as suspected drugs are shown in Figure 1. For both drugs we used the line listing function and searched for the following Preferred Terms (PTs): infusion related hypersensitivity reaction; infusion related reaction; injection related reaction; immediate post-injection reaction; anaphylactic reaction; anaphylactic shock; anaphylactoid reaction; anaphylactoid shock; influenza like illness; pyrexia. The search was made for years 2021–2023 (until November 3rd). ICSRs were downloaded as an excel file that was used to performed the descriptive analyses.

### 2.3 Descriptive analyses

Information on patient characteristics [age group (2 months—2 years, 12–17 years, 18–64 years and 65–85 years) and sex], AE (type, outcome and seriousness), therapeutic indication, primary source qualification, primary source country for regulatory purposes, number of suspected drugs other than ocrelizumab/ofatumumab, and number of concomitant drugs was provided for all ICSRs. To detect the use of a premedication, each ICSR was searched for the presence of corticosteroids, antihistamines and acetaminophen among suspected and concomitant medications and for the therapeutic indications “Allergy prophylaxis” or “Premedication.”

The seriousness degree was defined according to the ICH E2D guidelines (19). Thus, a “serious” case included ADRs that were life threatening, resulted in death, required or prolonged hospitalization, resulted in persistent or significant disability/incapacity, determined a congenital anomaly/birth defect, or resulted in some other clinically important conditions. The outcome was classified as favorable (“Recovered/Resolved” and “Recovering/Resolving”), unfavorable (“Recovered/Resolved with Sequelae,” “Not Recovered/Not Resolved,” “Fatal”) and not reported (“Unknown”). Lastly, the time to event (TTE) was calculated only for ICSRs that reported both the duration of the therapy and the drug withdrawal or the dose reduced as action taken after the occurrence of the ADR.

Data were analyzed using Microsoft Office Excel program. Box plots were done using STATA 18.

### 2.4 Ethical standards

Safety data extracted from the spontaneous reporting system comply with ethical standards and are anonymous. Therefore, no ethical measures were enforced further.

## 3 Results

### 3.1 Overall results

A total of 860 ICSRs were retrieved from Eudravigilance, of which 441 associated with ofatumumab and 419 with ocrelizumab. As reported in Table 1, the majority of patients who experienced ocrelizumab-induced IRRs belonged to the age group of 18–64 years, while the age-group was mostly not specified in ICSRs reporting ofatumumab as suspected drug. The distribution of gender was almost similar in the two groups, with the majority of ICSRs related to female patients. Similarly, no relevant differences were found between ICSRs related to ocrelizumab and ofatumumab in terms of the primary source qualification (mainly represented by Health Care Professionals) and for the majority of ICSRs, ofatumumab and ocrelizumab were the only suspected drugs (Table 1).

**Table 1:** Demographic characteristics and distribution of individual case safety reports (ICSRs) reporting ocrelizumab or ofatumumab as suspected drugs and PTs related to injection/infusion related reactions (IRRs) by primary source, number of suspected drugs other than ocrelizumab/ofatumumab and number of concomitant drugs.

Variable	Level	ICSRs with ofatumumab (*n* = 441)	ICSRs with ocrelizumab (*n* = 419)
Age group	2 months—2 years	–	1 (0.2)*
	12–17 years	1 (0.2)	2 (0.5)
	18–64 years	187 (42.4)	304 (72.6)
	65–85 years	10 (2.3)	5 (1.2)
	Not specified	243 (55.1)	107 (25.5)
Sex	Female	298 (67.6)	289 (69.0)
	Male	128 (29.0)	118 (28.2)
	Missing	15 (3.4)	12 (2.9)
Primary source	Healthcare professional	338 (76.6)	320 (76.4)
	Non-healthcare professional	103 (23.4)	99 (23.6)
Suspected drug(s) other than ofatumumab/ocrelizumab	1	10 (2.3)	78 (18.5)
	2	1 (0.2)	40 (9.5)
	3	–	7 (1.7)
	4	–	1 (0.2)
	≥5	–	8 (1.9)
Concomitant drug(s)	0	392 (88.9)	232 (55.4)
	1	27 (6.1)	38 (9.1)
	2	9 (2.0)	30 (7.2)
	3	5 (1.1)	29 (6.9)
	4	3 (0.7)	19 (4.5)
	5	–	15 (3.6)
	≥6	5 (1.1)	56 (13.4)

*Exposure via breast milk.

### 3.2 IRRs’ signs and symptoms, premedication and TTE

**Figure 2:**
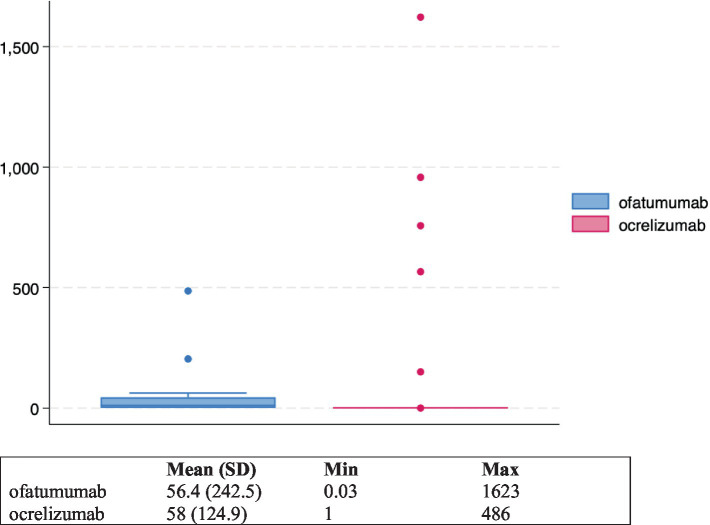
Box plots of median time to event in days for ocrelizumab and ofatumumab-induced IRRs.

As shown in Table 2, “Influenza like illness” and “Pyrexia” were most frequently reported with ofatumumab, while “Infusion related reaction” and “Anaphylactic reaction” were the most common reported PTs with ocrelizumab. The screening of ICSRs for the presence of premedication drugs (reported either as concomitant or suspected drugs) revealed the presence of such medications in 148 ICSRs, of which 131 related to ocrelizumab and 17 to ofatumumab (data not shown). Specifically, as reported in Table 3, the use of premedication drugs was more commonly reported in ICSRs describing cases of infusion related reaction (57% of all cases) and pyrexia (30.2% of all cases) (Table 3). Lastly, 89 ICSRs reported both the duration of the therapy and the drug withdrawal or the dose reduction as action taken after the occurrence of the ADR (73 related to ocrelizumab and 16 related to ofatumumab); thus, for these ICSRs, the TTE in days was calculated (Figure 2). The mean TTE was 56.4 days (Standard Deviation – SD: 242.5) for ofatumumab and 58 days (SD: 124.9) for ocrelizumab. Out of 89 ICSRs for which the TTE was calculated, 74 reported IRRs that occurred the same day of the DMT administration.

**Table 2:** Distribution of preferred terms (PTs) related to infusion/injection reactions by suspected drug.

	Ofatumumab (n, %) (*n* = 441)	Ocrelizumab (n, %) (*n* = 419)
Anaphylactic reaction	5 (1.1)	29 (6.9)
Anaphylactic shock	2 (0.5)	6 (1.4)
Anaphylactoid reaction	–	3 (0.7)
Influenza like illness	117 (26.5)	17 (4.1)
Infusion related hypersensitivity reaction	–	2 (0.5)
Infusion related reaction	4 (0.9)	225 (53.7)
Injection related reaction	9 (2.0)	1 (0.2)
Pyrexia	304 (68.9)	136 (32.5)

**Table 3:** Individual case safety reports (ICSRs) reporting cases of injection/infusion related reactions (IRRs) and suspected or concomitant drugs indicated as premedication agents.

ICSRs reporting IRRs (*n* = 860)	ICSRs reporting a premedication (*n* = 148)
Infusion related reaction (229)	84 (56.8)
Pyrexia (440)	45 (30.4)
Influenza like illness (134)	9 (6.1)
Anaphylactic reaction (34)	6 (4.1)
Anaphylactic shock (8)	2 (1.4)
Anaphylactoid reaction (3)	1 (0.7)
Injection related reaction (10)	1 (0.7)
Infusion related hypersensitivity reaction (2)	0 (0.0)

## 4 Discussion

To our knowledge, this is the first study that has compared infusion and injection reactions, including their potential symptoms, between ofatumumab and ocrelizumab through the analysis of data from the European spontaneous reporting system.

The main driver of targeting B cells in MS was based on the recognition of abnormally produced antibodies in the central nervous system of patients with MS (20, 21). Indeed, the increase in B cells in the cerebrospinal fluid of MS patients is positively associated with intrathecal inflammation and Ig synthesis (22). The availability of anti-CD20 monoclonal antibodies and the demonstration of their efficacy as selective B-cell-depleting therapies led to the recognition of the key role of B and T cell interactions in MS pathogenesis (23, 24). Monoclonal antibodies targeting CD20 deplete it through different mechanisms, including antibody-dependent cellular cytotoxicity, complement-dependent cytotoxicity, antibody-dependent cellular phagocytosis and induction of cell apoptosis (25). After their administration, the depletion of CD20+ B cells is observed within hours, mainly in the liver, reaching the nadir after 8 weeks and sustained for several weeks to months (26). As previously reported, among anti-CD20 drugs, those approved for the treatment of MS include ocrelizumab, which received the marketing authorization by the EMA in 2018 for the use at a dose of 600 mg to be administered intravenously twice yearly (27), and ofatumumab that is, instead, authorized in a formulation to be subcutaneously injected. Compared with the intravenous administration, the subcutaneous one should lead to more efficient and selective targeting of B cells in the lymphatic circulatory system and isolate the drug into the hypodermis, preventing its spread into the systemic circulation (9, 28).

In our study we described the main characteristics of 860 ICSRs reported to the EV database during years 2021–2023, of which 441 related to ofatumumab and 419 related to ocrelizumab, and reporting cases of IRRs. We found that the majority of these ADRs occurred in female patients aged 18–64 years. These results are not surprising. Indeed, MS mainly affects adult women (29). Moreover, there are also gender differences in the frequency of RRMS forms (females are more susceptible than males in experiencing relapses) (30). In addition, apart from sex-differences in MS prevalence and progression, ADRs in general occur more commonly in women due to sex-related factors affecting pharmacokinetic and pharmacodynamic processes that in turn affect the safety profile of drugs (31). For instance, the pharmacokinetic processes diverge between women and men due to a different expression of metabolic enzymes and to a reduced renal clearance of drugs in females because of a lower glomerular filtration rate compared to males (32, 33). These differences could explain the increased rate of ADRs that is generally observed in women compared to men. There is also a gender difference in reporting ADRs; as a matter of fact, compared to men, women show greater interest in reporting ADRs and tend to report more detailed information (34, 35).

As reported by Florou et al. (7), cytokine release makes IRRs the main AEs occurring after the administration of anti-CD20 monoclonal antibodies, especially after the first infusion, with symptoms that include urticaria, angioedema, headache, nausea, fever, chills, and rarely bronchospasm. This is in line with our results related to the most commonly reported PTs but also those related to the TTE suggesting that the risk of IRR is higher immediately after the first infusion (even though this last result was presented for a limited number of ICSRs).

Although we have not found a substantial difference in the number of ICSRs reporting IRRs between ofatumumab and ocrelizumab (441 vs. 419), looking at IRRs’ signs and symptoms a difference between these drugs can be found. For instance, almost 70% of ICSRs related to ofatumumab reported cases of pyrexia, while more than half of ICSRs related to ocrelizumab reported cases of IRRs (reported as PT). Anaphylactic reactions were more frequently reported with ocrelizumab than ofatumumab (6.9% vs. 1.1%, respectively). This is in line with data from phase III trials that suggested that IRRs occur with a lower incidence with ofatumumab (20.2%) compared to ocrelizumab (34.3%) (36). Specifically, safety data derived from OPERA I and II studies reported that IRRs occurred in 34% of the RRMS patients treated with ocrelizumab compared with 10% of those treated with INFβ-1a or placebo. Similarly, data from the ORATORIO trial reported a prevalence of IRRs equal to 40% with ocrelizumab and 26% with placebo in PPMS patients (37). On the other hand, safety data from ASCLEPIOS I/II ALITHIOS studies reported no difference, after the first injection, in frequency and severity of IRRs between ofatumumab and teriflunomide groups (10, 13), even though among AEs of special interest (AESIs) identified for ofatumumab there were systemic injection-related reactions (9, 10). In particular, symptoms of systemic injection-related reactions, which occurred in almost 14% of patients at the first injection and in <3% of patients from the third injection onward, included fever, headache, myalgia, chills and fatigue. IRRs were generally mild to moderate in severity (only 2 cases were classified as grade 3) and did not require treatment (only one patient discontinued the treatment due to the IRR). Recently Zanghì et al. (38) carried out a retrospective cohort study among 396 MS patients who initiated treatment with ocrelizumab (*n* = 216) of ofatumumab (*n* = 180) at eight Italian MS centers and with at least 12 months lasting therapy. In line with previously reported data, patients receiving ofatumumab mainly experienced flu-like syndrome at the first administration with early resolution. Upper respiratory tract infections were reported in a slightly higher percentage of patients receiving ocrelizumab compared to those initiated with ofatumumab (13.8% vs. 11.1%). Headache was the second most common AE in the ocrelizumab group.

Since IRRs are recognized as common ADRs associated with ocrelizumab intravenous infusion, a premedication with antihistamine drugs, acetaminophen or glucocorticoids, is highly recommended to prevent their occurrence. As reported in the summary of product characteristics (27), the premedication treatment includes the administration of: 100 mg intravenous methylprednisolone 30 min prior to each infusion; antihistamine 30–60 min prior to each infusion; a premedication with an antipyretic may also be considered 30–60 min prior to each infusion (27). As previously reported, the premedication is not recommended before ofatumumab subcutaneous injection (9). In line with this, we found concomitant or suspected drugs used as premedication in 131 ICSRs related to ocrelizumab vs. 17 ICSRs related to ofatumumab.

Lastly, the majority of ICSRs retrieved for both suspected drugs were reported by healthcare professionals. This is in line with previous studies carried out on data from spontaneous reporting systems (31, 39, 40). Nowadays patients are increasingly covering a proactive role in the collection of spontaneous reports of ADRs. Indeed, patients’ contribution to pharmacovigilance systems is undeniable considering that reports coming from drugs’ users bring their unique perspectives and experiences as well as information that are generally not provided by HCPs, including suspected reactions to over-the-counter medicines and different presentations of reactions, including those affecting patients’ quality of life (41). Notwithstanding this, HCPs still represent the main report of ADRs, especially serious ones, playing a crucial role in characterizing safety data during the pharmacovigilance surveillance, helping the identification of new potential AEs. In particular, the systematic review carried out by Inácio et al., and other studies as well, suggest that healthcare professionals tend to report more frequently serious events rather than not serious ones (42).

Our study has many strengths. First of all, we used data from the European spontaneous reporting system which collects ICSRs spontaneously reported by citizen/patients and HCPs reflecting the real-world experience with drugs. This represents an added value because information gathered with spontaneous reporting system cannot be easily examined during premarketing clinical studies due to ethical/methodological considerations (frail population are excluded from the classic RCTs, the study’s duration is limited and does not allow to detect long-term or rare ADRs, etc.). In addition, in the context of spontaneous reporting databases, the EudraVigilance represents the largest pharmacovigilance database that gathers heterogeneous data from many demographics and nations (43).

Our study has many limitations too. First of all, spontaneous reports often lack of demographic and clinical data. For instance, the age group was missing in 350 ICSRs. In addition, although the premedication is recommended before ocrelizumab infusion, drugs used for this indication were reported only in 31% of ICSRs reporting ocrelizumab as suspected drug, suggesting again that ICSRs are not properly filled out. Second, we should take into account the under-reporting that represents the main intrinsic limitation of spontaneous reporting systems, being one of the major disadvantages of pharmacovigilance methods that can lead to an underestimation of the frequency of some ADRs and hide potential drug related risks. Moreover, information about drug withdrawal or drug reduction and therapy duration were not available in the majority of ICSRs. Thus, we were able to calculate the TTE only for 89/860 ICSRs, suggesting that this result should be interpreted carefully.

## 5 Conclusion

The analysis of data reported in the EV database showed a similar number of ICSRs reporting IRRs following ofatumumab and ocrelizumab treatments although few differences were noted in terms of IRRs’ signs and symptoms. Indeed, ofatumumab was mainly associated with the occurrence of pyrexia while ocrelizumab was mainly reported as suspected in cases of infusion related reactions (reported as PT). Thus, although a risk of ofatumumab-induced IRRs cannot be excluded, it should be considered as manageable considering that the drug seems to be mostly associated with the occurrence of fever. Nevertheless, MS patients should be properly informed of the possibility of IRRs’ signs and symptoms following the subcutaneous administration of ofatumumab, which as any other mAb, can be also related to the occurrence of serious infusion reactions (44). Thus, the monitoring of DMTs, especially those that were recently approved, is highly recommended in order to better characterize their safety profile. Indeed, the collection and analysis of new data on this potential serious consequence of the subcutaneous administration of ofatumumab might lead to a new knowledge that could potentially bring to the development of new guidelines that can guide neurologists in their daily activities when administering this drug.

## Data availability statement

Publicly available datasets were analyzed in this study. This data can be found at: European Medicines Agency (EMA), EudraVigilance (https://www.adrreports.eu/en/search.html).

## Ethics statement

Ethical approval was not required for the study involving humans in accordance with the local legislation and institutional requirements. Written informed consent to participate in this study was not required from the participants or the participants' legal guardians/next of kin in accordance with the national legislation and the institutional requirements.

## Author contributions

CS: Conceptualization, Investigation, Methodology, Validation, Writing – original draft, Writing – review & editing. AA: Conceptualization, Investigation, Methodology, Writing – original draft, Writing – review & editing. IB: Investigation, Writing – review & editing. ACan: Investigation, Writing – review & editing. DG: Formal analysis, Investigation, Writing – review & editing. FB: Formal analysis, Writing – review & editing. OM: Formal analysis, Investigation, Writing – review & editing. VL: Formal analysis, Methodology, Writing – review & editing. VA: Conceptualization, Data curation, Writing – review & editing. GM: Conceptualization, Data curation, Supervision, Writing – review & editing. ACap: Conceptualization, Data curation, Project administration, Supervision, Writing – review & editing.
